# Relationship between Moderate-to-Vigorous Physical Activity with Health-Related Physical Fitness Indicators among Pakistani School Adolescents: Yaali-Pak Study

**DOI:** 10.1155/2022/6402028

**Published:** 2022-09-07

**Authors:** Syed Muhammad Zeeshan Haider Hamdani, Zhuang Jie, Syed Ghufran Hadier, Wang Tian, Syed Danish Haider Hamdani, Shaista Shireen Danish, Syeda Urooj Fatima

**Affiliations:** ^1^Faculty of Sport Science, School of Kinesiology, Shanghai University of Sport, Shanghai, China; ^2^Faculty of Arts & Social Sciences, Department of Sports Sciences, Bahauddin Zakariya University, Multan, Pakistan; ^3^School of Physical Education, Shanxi University, Taiyuan, China; ^4^School of Sports Science, Beijing Sport University, Beijing, China; ^5^Department of Physical Education and Sports Sciences, Government College University Faisalabad, Faisalabad, Pakistan

## Abstract

**Background:**

The current study is the foremost study exploring the relationships between moderate-to-vigorous physical activity and health-related physical fitness indicators among 12–16-year-old adolescents of the South Punjab region of Pakistan.

**Methods:**

The researcher adopted the cross-sectional research design for the study. A total of 2970 participants (1477 boys and 1493 girls) aged adolescents from South Punjab, Pakistan, completed health-related physical fitness indicators measuring strength, endurance, and aerobic capacity through a hand-grip strength test, modified pull-up test, plank test, and 20-m shuttle run test, and physical activity were subjectively assessed by International Physical Activity Questionnaire Short Form (IPAQ-SF). Linear regression models were used to explore the between moderate-to-vigorous physical activity (MVPA) with health-related physical fitness indicators.

**Results:**

Positive associations were observed between hand-grip strength (*p* < 0.001), modified pull-up (*p* < 0.001), plank exercise (*p* < 0.001), and 20-m shuttle run test (*p* < 0.001), with MVPA. The gender-specific comparison also indicated a significant (*p* < 0.001) and positive relationship. The results revealed that as MVPA increases, body composition, muscular strength, core muscular endurance, and aerobic capacity could improve in both genders.

**Conclusions:**

MVPA appears to be an effective and reliable predictor of health-related physical fitness among school adolescents.

## 1. Introduction

Recently, increased interest has been shown by the researcher and investigators in the prevalence of adiposity, inactivity, and health risk factors among adolescents, and the appearance of cardiovascular, neuromuscular, and metabolic diseases during adolescence emphasized the necessity to consider physical activity and physical fitness vital in the growth of adolescent children [1]. It is reported that physical activity is effectively protected against numerous diseases such as hypertension, metabolic syndrome, diabetes type II, and obesity [2]. Physical fitness is an effective predictor of good health and helps individuals prevent obesity in adolescents [3]. Researchers have emphasized that obesity in adolescents can be prevented by acknowledging its inverse relationship with physical activity [4].

### 1.1. Physical Fitness Definition and Lacking Reasons

Physical fitness is a valid indicator in determining the adolescent's health status [5]. Physical fitness is divided into two categories: health-related physical fitness and skill-related physical fitness. The assessment of general health status and health-related physical fitness capacities (aerobic fitness, muscular strength, muscular endurance, flexibility) is widely used among all ages [6]. Physical fitness among school-aged adolescents has been observed to decline in the last few decades as well as physical activity levels are also not up to recommended standards internationally, ultimately creating an alarming situation [7–10].

### 1.2. PA Definition and Lacking Reasons

Physical activity is defined in a variety of aspects such as the inherent aspect of PA, clinical, educational, research, and policy settings. Initially, physical activity is defined narrowly as the human body movement produced by the skeletal muscles. The evolved definition of physical activity from the researchers defines physical activity as the movement, actions, and activities of people in specific places and cultural contexts and is influenced by a series of interests, feelings, thoughts, attitudes, and behaviors [11]. Overall, public values, cultural constraints, environmental conditions, access to public parks, sports facilities, family structures, and sedentary behavior reflected the decline in physical activity, specifically among adolescents.

PF and PA in adolescents' importance: Sufficient physical activity is significantly essential and provides the foundation for physical fitness in adolescents and later age. Elevated PA levels, specifically moderate-to-vigorous physical activity (MVPA), are associated with higher fitness levels, bone density, heart and lung function, and adiposity prevention among adolescents [12]. Although the medical symptoms of heart disease appear later in life, it is known that the occurrence of cardiovascular diseases starts at an early age; thus, preventive measures, including a healthy lifestyle, should be adopted in childhood [13]. However, it is evident scientifically that a positive link exists between physical activity and health-related physical fitness in adolescence [14].

### 1.3. Effect of PA and PF on Physiological and Psychological Development

Rodriguez-Ayllon stated that physical activity was inversely related to mental illness (e.g., depression, stress, anxiety, and overall anatomical distress) and positively related to mental well-being (e.g., self-esteem, life satisfaction, happiness, and mental health). That is why research indicated that increased physical activity reduces sedentary behavior and might improve the mental health of adolescents [15]. Higher physical activity levels, specifically vigorous activities, are significantly associated with cardiovascular fitness, movement skills, and less percentage of lipids in the body [16]. The dynamic growth and development that occurs at a young age may be the explanation for this vibrant relationship. The fitness level of adolescent and early aged population changes while making performance with the change in the levels of physical activity, but notably, the direction of this relationship is ambiguous [17, 18]. At the same time, it is crucial to understand the deep connection between physical activity behaviors and early childhood physical fitness in order to develop effective programs for this group. Early childhood is a significant period in the development of a person's life in the development of positive and healthy attitudes, including the level of physical activity from early childhood to adolescence and lifelong benefits of physical fitness [1, 19]. These outcomes illustrated the benefits of physical activity and physical fitness involvement in early life.

At this time, limited information is known regarding the relationship between physical activity and health-related fitness, according to the researcher, among school-aged adolescents globally, but no prior study was published on the said population in Pakistan. Hence, The purpose of this study was to evaluate the cross-sectional connection between subjectively perceived physical activity and health-related physical fitness indicators among the school-going adolescents in South Punjab, Pakistan.

## 2. Materials and Methods

The researcher adopted the cross-sectional research design for the study. Adolescents of South Punjab, Pakistan, participated in moderate-to-vigorous physical activity (MVPA) and health-related physical fitness Young teens Assessment Active Lifestyle Involvement-PAKistan (YAALI-Pak) study. The researcher implemented stratified random sampling to obtain the purpose of the current cross-sectional study [20]. South Punjab province was selected as strata, which were divided into three strums: Multan, Bahawalpur, and D.G. Khan. South Punjab province contains 485 public schools, out of which 12% (60 public high schools) of high schools were randomly selected from each strum on an equal allocation basis (20 high schools from each strum) [20]. The data collection was completed in 2019 with the approval of district management and higher education authorities. The sample size was specified by the commonly used following equation for the determination of sample size by the researcher [20, 21]:

(1) $$\begin{array}{c} n=\frac{{\left(Z\right)}^{2}PQ}{e^{2}}\times D, \end{array}$$

(2) $$\begin{array}{c} n=\frac{{\left(1.96\right)}^{2}\left(0.4\right)\left(0.6\right)}{{\left(0.0175\right)}^{2}}\times 1=3011\approx 3012. \end{array}$$

A sample size of 3012 was deliberate from (2) with the values of *P*=0.4 (40%), *Q* = 0.6 (1-P), Z-score = 1.96 at 5% significance level, *e* = 0.0175 level of precision, and *D* = 1 (*D* is the design effect) [21]. Therefore, 3012 adolescent boys (50%) and girls (50%) were recruited as a sample for the study. After removing forty-two outliers, a total sample of 2970 school adolescents, boys 1477 (49.73%), and girls 1493 (50.26%), were analyzed for the study. Written and verbal consent was obtained from the local government authorities, educational authorities, school management, caretakers, and parents. Two workshops were conducted prior to the sampling and testing procedure, one with the school management and parents and the other with the volunteered test and measurement staff and students. During the workshops, all necessary information regarding the study project and its testing phase were delivered to concerning parties. Physically and mentally healthy adolescents with no previous injury record or monetary reimbursement intention were voluntarily included in this study. The current research design and methodology were approved in September 2018 by the School of Kinesiology, Faculty of Sport Sciences, Shanghai University of Sport, and Institution Review Board from the Ethics Advisory Committee of Shanghai University of Sport, Shanghai, China.

### 2.1. Measures and Procedures

The study followed the protocol for MVPA assessment from IPAQ, and health-related physical fitness indicators were followed by National Youth Fitness Survey (NYFS) by the Centers for Disease Control and Prevention, USA protocol, and fitness Gram. Before starting the physical fitness tests, sufficient time was provided for the subjects to warm up, and all the subjects were also recommended to perform cool-down exercises to avoid any possible injury.

### 2.2. Selected Anthropometric Parameters and Body Mass Index

Anthropometric characteristics were measured by following the procedures described in the literature. The measurements of individuals were documented bare-footed, wearing simple and light clothes to avoid false calculations. A height measuring scale and portable digital weight machine were used to measure, and the BMI was calculated mathematically from the height and weight values.

### 2.3. Health-Related Physical Fitness Measures

#### 2.3.1. Core Muscular Endurance Plank Exercise (PE)

Plank is an isometric exercise test that is conducted to assess core muscular endurance [22].

#### 2.3.2. Test Method

The plank test protocol requires the participants to hold or maintain the static horizontal position as long as the subject can without getting easily fatigued or injured. During the test, the tester holds a stopwatch to start timing. The subject lies prone on the mat and bends their elbows. The shoulder and elbow joints were perpendicular to the ground. The feet stepped on the ground, the body was off the ground, the trunk was straight; the head, shoulder, hip, and ankle were kept in the same plane, the abdominal muscles were tightened; the pelvic floor muscles were tightened and the spine was extended; the eyes were looking at the ground; and the breathing was maintained evenly. The test is finished if the subject's action deviates from the standard action during the test. The tester reported the time of the subject to the recorder, and he recorded it in seconds.

#### 2.3.3. Reliability and Validity of Tool

In previous years, numerous researchers had adopted plank exercise to measure core muscle endurance, proving itself as a valid, reliable, and practical measurer [23].

#### 2.3.4. Upper Body Muscular Strength Hand Grip (HG)

Grip strength is mainly used to test upper-limb muscle strength, reflecting the development level of an adolescent's upper-limb strength.

#### 2.3.5. Test Method

A standard GRIPX Digital Hand Dynamometer Grip Strength Measurement was used to measure hand-grip strength. Before the test, use the handle adjustment knob to adjust the handle distance according to the palm-size. Hand-grip strength was observed in the subject when he was in a standing position with naturally separated feet and shoulders in an adducted position, and was neutrally rotated as well as the arms of the subjects were naturally opened 30 degree' parallel but not in touch with the body interface. The students were asked to squeeze the handle for 3–5 seconds. The device LCD showed the numeric values as a result; furthermore, it was recorded by the recorder. The researcher elaborates the detailed procedure for the HG measurement [21, 24].

#### 2.3.6. Reliability and Validity of Tool

The literature shows that the hand-grip test is a reliable and valid test to measure the status of an individual's upper body muscular strength, as reported in [24].

#### 2.3.7. Muscular Strength Modified Pull-Up (MPU)

Modified pull-up is a hanging strength test used to assess the body's muscular strength. It is the most basic way to exercise back and strength testing.

#### 2.3.8. Test Method

The modified pull-up was conducted with a modified horizontal tow bar and gymnastic mat. The modified horizontal bar was placed on the flat ground and positioned at a height that allows the participant to clasp the bar with an overhand grasp when lying on his/her back on a flat surface. Before the test, the modified tow bar was adjusted to the appropriate height, and the gymnastic mat was placed under the tow bar. During the trial, a nylon strap hung 8 inches down from the center of the horizontal bar. The participant was asked to hook the bar with an overhand grasp of both hands the same width as their shoulders and palms facing away from the body. Push their feet forward to the ground, keep their body straight (the angle between the body and the ground is less than 45 degree), hang their arms at 90 degree to the trunk, bend their arms to make their mandible touch or exceed the crossbar, and then, straighten their arms to recover. Each time the subjects reached the standard action, it was recorded as one. The test was stopped when the participant paused for two or more seconds, could no longer maintain the correct position, or requested the test be stopped. Scores were based on the number of pull-ups completed correctly.

#### 2.3.9. Reliability and Validity of the Tool

A study by Ortega et al. provides evidence that the modified pull-Up test is a reliable and valid test to measure the status of muscular strength in adolescents [5].

#### 2.3.10. Aerobic Capacity Shuttle Run Test (SRT 20 m) Completed Laps

The participant's aerobic capacity was measured through the shuttle run test of 20 m. Adolescent students need to run back and forth across a marked 20 m track during the test considering the time to start with beeps every time. The individuals started running at the speed of 8.0 km/h; at the next stage, it was at 9.0 km/h, and the speed increase was recorded by 0.5 km/h each minute. The participants were required to reach across the line before the beep. If an individual is unable to reach the any of the endpoints before blowing of beep sound, he was warned for the first time, and the second time if he did so, he was disqualified or out from the test/race, and the completed laps were considered his result and noted by the recorder.

#### 2.3.11. Reliability and Validity of the Tool

Mayorga-Vega et al provided a meta-analysis on the criterion validity of the shuttle run test among adolescents to measure aerobic capacity; it showed moderate-to-high criterion-related validity (*r* = 0.66–0.84), and the study concluded that the shuttle run test is a valid test to provide the estimate of aerobic capacity. The literature shows that the SRT test is a valid test to measure an individual's aerobic capacity status [25].

### 2.4. Physical Activity Measures and Procedures

A team of well-trained research assistants administered the survey of physical activity according to the guidelines of short form of International Physical Activity Questionnaire (IPAQ). MVPA minutes per day were assessed from the selected items of International Physical Activity Questionnaire (IPAQ) short form. Children were given detailed instructions on how to fill out the survey and were provided ample time for questions. Students completed the survey form independently; proper assistance was provided to each student while filling in the information. In this survey, the provided data were self-reported voluntarily by the participants, with the exception of height and weight. The short form of IPQA, known as IPQA-SF, consists of 7 items in total, which measure the time spent in MVPA per day and the sedentary behavior sitting time spent per day and is widely used by researchers to be very useful for surveillance studies [26]. The researcher measured the PA level through the MVPA minutes, and the same method was adopted by [27]. In this method, MVPA minutes were calculated by moderate and vigorous activities in which they were involved seven days a week. The sum of both moderate activities and vigorous activities was divided by seven. After acquiring MVPA minutes, they were categorized into the meeting and nonmeeting categories of PA recommendation for 60 minutes per day of physical activity.

### 2.5. Statistical Analysis

Following the collection of raw data, we gave each person a unique identification number that represented their gender and area status (male = 1, female = 2). After using Microsoft Excel, the data were entered into a spreadsheet. Following the identification of the outliers, a final sample consisting of 2,970 participants, which represents about 98% of the total selected population, was chosen for additional statistical analysis. Descriptive analysis was performed using Statistical Package for the Social Sciences (SPSS) version 21.0 to examine percentages (%), frequencies, mean (average), and standard deviation (SD). The current study intended to discover the relationship between MVPA and components of health-related physical fitness among school adolescents of Pakistan. Linear regression analysis was performed to find the relationship between selected health-related physical fitness components and moderate-to-vigorous physical activity (MVPA min/day) for adolescents. The current study includes components of health-related physical fitness as a dependent variable and MVPA as an independent variable in linear regression analysis.

## 3. Results

Table 1 shows the population's age- and gender-specific demographic and geographic descriptive analysis. The current study testified a total of 2970 participants (12–16-year-old school adolescents). Boys and girls were distributed equally as 1477 (49.7%) and 1493 (50.3%). As per the residential area, the population from each strum was equally allocated as 990 (33%). Furthermore, around 20% of the population was distributed from each age group with each gender.

Table 2 shows the gender-specific descriptive analysis of the anthropometric measures and physical fitness components. The average age, height, weight, and BMI values were higher in boys (14.01 ± 1.41, 160.50 ± 11.50, 45.02 ± 9.78, and 17.30 ± 2.41) than in girls (14.00 ± 1.41, 158.57 ± 9.34, 41.00 ± 7.89 and 16.29 ± 2.82). On the other hand, similar findings can also be observed in physical fitness indicators, that is, HG, PE, MPU, and SRT, where boys had higher average values (31.81 ± 14.85, 80.72 ± 73.48, 12.53 ± 7.74, and 31.00 ± 17.00) than girls (17.06 ± 5.39, 69.07 ± 58.16, 5.06 ± 4.69, and 23.00 ± 14.00). Average MVPA values were the same in both genders as 58.05 ± 37.02. While comparing the gender with respect to anthropometric measures and physical fitness indicators, the results were found to be significant (*p* < 0.05) among components of physical fitness (HG, PE, MPU, and SRT (*L*) except PE. Overall, the table showed that boys had higher average values in physical fitness than girls. In contrast, while comparing the gender with respect to physical activity, the results were not significant (*p* > 0.05) with MVPA.

Differential associations between MVPA and health-related physical fitness components are presented in Table 3 using simple linear regression analyses for both genders. It showed that there was a significant relationship between health-related physical fitness indicators with moderate-to-vigorous physical activity, whereas regression coefficient (*β*), coefficient of determination (*R*^2^) and significant value (*p* < 0.001) of indicators such as health-related physical fitness indicators as the hand grip (*β* = 0.297, *R*^2^ = 0.547, *p* < 0.001), modified pull-ups (*β* = 0.106, *R*^2^ = 0.411, *p* < 0.001), plank exercise (*β* = 0.894, *R*^2^ = 0.382, *p* < 0.001), and 20-m shuttle run (*β* = 0.332, *R*^2^ = 0.531, *p* < 0.001) were found positively associated with MVPA. No negative relationship was found between any indicators of health-related physical fitness with MVPA, similarly reported by the HELENA study [28].

Tables 4 and 5 detail the cross-sectional associations between MVPA and health-related physical fitness indicators discretely using multiple linear regression analyses in boys and girls, respectively.

However, in both boys and girls, there was a positive relationship found between health-related physical fitness indicators as the hand grip (*β* = 0.387, *β* = 0.21, *R*^2^ = 0.577, *R*^2^ = 0.665, *p* < 0.001), modified pull-ups (*β* = 0.151, *β* = 0.063, *R*^2^ = 0.499, *R*^2^ = 0.403, *p* < 0.001), plank exercise (*β* = 0.956, *β* = 0.833, *R*^2^ = 0.364, *R*^2^ = 0.413, *p* < 0.001), and 20-m shuttle run (*β* = 0.386, *β* = 0.279, *R*^2^ = 0.556, *R*^2^ = 0.526, *p* < 0.001) with MVPA.

## 4. Discussion

It is the first time that any information has been published on the association between adolescents' health-related physical fitness, physical activity, and sedentary behavior in the south Punjab province of Pakistan. To the best of our knowledge, this is the first article to precisely examine health-related physical fitness, physical activity, and sedentary behavior with these indicators and tools; therefore, our findings cannot be compared to those found in the local (Pakistani) literature. Several studies have proved that MVPA and physical fitness benefit one's health lifetime [29–31].

### 4.1. BMI

The current study found that 14.52% of the total population was falling under the obese and overweight category of BMI; however, girls' percentage of obesity was higher than boys. The literature suggests that a person's body mass index (BMI), physical activity, and fitness level can be predicted by their sexual orientation, age, height, and weight. However, the impact of these predictors differs based on the test batteries utilized. On the other hand, findings demonstrate that BMI strongly correlates with health-related physical fitness, physical activity, and sedentary behavior. The current study assessed the BMI of the participants as well.

Moreover, current research is a part of the YAALI-PAK study, which also separately estimated the BMI relationship with PA and PF. A study discovered a relationship between vigorous physical activity, cardiorespiratory fitness, and BMI [32]. In younger children and adolescents, there is an inverse association between body composition (BMI) and aerobic fitness [33, 34]. According to Gaylis et al., most adolescents, particularly females, have a distorted perception of their body weight, which may impact their desire to lose weight. Misperceptions about body weight were seen across nationalities and BMI categories. The fact that adolescents who believe they are skinny consume more unhealthy meals than those who believe they are overweight is particularly troubling [35].

### 4.2. Upper Body Strength (HG)

Tests of upper- and lower-body strength in adolescents have demonstrated that hand-grip strength has moderate-to-good validity and excellent reliability. Furthermore, the validity, reliability, and practicality of hand-grip strength have been established in educational settings [36, 37]. Bone density can be boosted by a combination of high-intensity physical activity and strength training. Bone density can be improved with regular, high-intensity exercise. Strength training can help preserve bone density by increasing blood PH and changing the biochemistry of free ionized calcium, in addition to the mechanical strain it places on the bones [38]. The current study showed a positive and significant relationship between strength (hand grip, modified pull-ups) and endurance (plank) with moderate-to-vigorous physical activity. Huang and Malina conducted a study on Taiwan's adolescent's age group and concluded a significant relationship with a low-correlation between physical activity and the physical fitness components of strength and endurance [39]. In older individuals, hand-grip strength is a general indicator of bodily strength. Strength by hand grip and balancing skills jointly expounded 31% of the variance in the total quality of life, according to DPA multiple linear regression analysis [40]. A study conducted among Kenyan adolescents, on the other hand, discovered that female adolescents have greater muscle strength than their males. The researchers hypothesized that possibly, this was because women were more responsible for domestic tasks like moving water fetching from deep wells and cooking meals, which meant they had more accessible access to food than men [41]. Over time, these activities aid in the growth of upper-limb muscle mass and power.

On the other hand, these circumstances are not typical in Malaysia, where the vast majority of adolescents are school-going. As a result, it is assumed that the reason behind low muscular strength among female adolescents in this study than male adolescents. According to the findings, approximately 70% of females had lower physical activity scores than their male counterparts because of their sedentary behavior, while 65% of the boys had high moderate to high physical activity score [36]. At the same growth stage, more significant male preadolescent muscle hypertrophy is more prevalent than female preadolescent muscular hypertrophy, owing to higher amounts of circulating androgens in males [42]. In addition, prior studies discovered [5, 36, 43] that girls begin to have lower physical activity scores at a younger age than boys and that this trend persists as they grow older. An additional potential explanation for this statistically substantial result encountered in boys but not in girls could be due to changes in the body during puberty. Male hand-grip strength rises substantially earlier than female grip strength in childhood.

There was a correlation between hand-grip strength and the physical activity score (*r* = 0.170; *p*=0.001) after controlling for ethnicity, place of residence, and body mass index (Ng, 2019). Body composition, cardiovascular capability, and muscular strength were all strongly correlated with reported physical activity levels. Additionally, the current study found a favorable and significant link between MVPA and the hand-grip and modified pull-ups test, with values of *β* = 0.297, *R*^2^ = 0.547, *p* < 0.00 and *β* = 0.106, *R*^2^ = 0.411, *p* < 0.00, respectively. Additionally, the current study found that boys had higher average levels of HG and MPU than girls. This is consistent with the long-held belief that increasing physical activity leads to an increase in strength and aerobic capacity, which has been shown previously [44].

### 4.3. Plank

Adolescents who participated in core strength training experienced significant improvements in physical fitness proxies [45]. Conducted on finding the relationship of PA with the physical fitness variables and motor skills, the results concluded that physical activity had a significant but low to moderate correlation with longer plank time (*β* = 0.182, *p*=0.001, *R*^2^ = 0.023) [46]. The current study found the plank test results of *β* = 0.894, *R*^2^ = 0.382, *p* < 0.001, indicating a positive and statistically significant association between MVPA and plank. Girls were found to have more average values on plank tests than boys; the results are inconsistent with the literature. Strong empirical evidence is lacking that physical activity rather than physical fitness or motor competence is of primary importance to health among children and adolescents [47, 48].

### 4.4. Aerobic Cardiovascular Fitness (20 m-SRT)

According to hands, physical activity and aerobic fitness are two distinct entities, and the association between them in this study was weak (*r* = 50.16 in males, *r* = 50.23 in females), indicating a degree of independence [49]. Others have experienced similar outcomes. For example, in younger children [50, 51], and adults, there was a weak to moderate correlation between physical activity and aerobic fitness [52]. The regression models validated these findings. In the study hands, only aerobic fitness was a significant predictor of physical activity among the physical fitness factors studied [49]. Following the findings of the regression models discussed above, aerobic fitness should be deemed to be of primary relevance for various reasons.

First and foremost, aerobic fitness was found to be associated with physical activity. Second, valid and reliable measures of aerobic fitness for children and adolescents are available globally, although physical activity measurement in this age group remains a challenge. While it is critical to instill in youngsters a healthy level of habitual physical exercise, it is controversial whether this is more important than emphasizing fitness [53]. Current research also supported the outcomes of previous studies; cardiovascular assessment by 20 m shuttle run test results reflected numerically as *β* = 0.332, *R*^2^ = 0.531, *p* < 0.001, and the relationship was found positive and significant with MVPA. Physical fitness is not understood as an umbrella that covers all aspects of fitness health but as a synonym for aerobic fitness. These findings highlight the importance of finding ideas for improving physical fitness. Participants who performed well on one fitness component did not always do well on another. To maintain a healthy fitness level, it appears important to engage in various physical activities at an appropriate intensity level that has not yet been determined [49].

According to the currently known research, physical activity (MVPA) and health-related physical fitness among adolescents appear to have a strong correlation. Health-related physical fitness was strongly associated with moderate-to-vigorous physical activity [54]. Independent of physical activity, measures of health-related physical fitness fluctuate with growth and maturation across the first two decades of life and with age throughout the adult years. Nonetheless, the patterns underscore the necessity of a lifestyle of regular physical activity throughout the adolescent years, which continues into adulthood, for the health and well-being of the person and the population [19]. The current study also showed similar results as earlier studies suggested that MVPA is a positive and significant relationship with health-related physical fitness. Our findings, however, indicate that the effect of moderate-to-vigorous physical activity (MVPA) on health-related physical fitness appears to be partially determined by the effect of physical activity on health-related physical fitness. Physical activity could help students develop and improve their physical fitness. We feel that while increased physical fitness does not ensure a much more active lifestyle, it is critical to developing moderate-to-vigorous physical activity habits across childhood to ensure a child's physical health.

### 4.5. Strength of This Study

Scientists have reached various conclusions due to differences in measurements, methods, populations, and contexts in connection to physical exercise and physical fitness. Growth factors may skew the findings of research conducted on children and adolescents around the time of puberty. Body proportions can significantly impact performance, but the ability to adapt to these changes is a skill that can be learned [50]. It is crucial to note that one of the major strengths of the current study is that it examined the relationship between moderate-to-vigorous physical activity with health-related physical fitness in adolescents in a cohesive approach. The gender difference and the relationship between various physical fitness tests and adolescent physical activity have also been taken into account. Additionally, adolescents' health-related physical fitness and moderate-to-vigorous physical activity levels were assessed comprehensively using sensitive process measures, and all analyses were adjusted for potential confounders.

### 4.6. Limitation of This Study

Despite this, one drawback of our study is that physical fitness evaluations were carried out in school settings, which may not be precise compared to laboratory tests using an epidemiological method. Fitness assessments conducted in more experimental settings may more accurately reflect performance levels in real-world situations. Apart from that, children in this particular age range may still be lacking in knowledge and coordination when participating in health-related physical fitness assessments. In order to determine whether there is a significant association between body composition, physical activity, and health-related physical fitness in this age range, additional research is required. Furthermore, the flexibility component of health-related physical fitness was not included in this study, as explained earlier by the US committee on fitness measurement and health outcomes in youth; the institute of medicine recommendation concluded that the relationship between flexibility and health was found vague due to the nonavailability of the scientific literature; thus, committee did not recommend including flexibility in national fitness surveys in the USA [55]. Therefore, following the recommendation mentioned above, the current study did not account for flexibility and kept flexibility limited. Another research in China by Fang states that in our study, no significant relationship between flexibility and physical activity was found in both genders. However, flexibility is an important component of health-related physical fitness; the current literature defined the relationship between physical activity and flexibility in children as “very limited” and only looked at older persons [56, 57].

Additionally, it is recommended to find the different relationships of BMI with the health-related physical fitness indicators and physical activity of the said age group and territory. Additionally, our sample is drawn primarily from three districts of the province of South Punjab. Further research is needed on the same age group on a large scale to generalize the whole population of Pakistan.

## 5. Conclusions

Public Health and Clinical Implications: in brief, this study provides the foremost and latest information on the relationship between moderate-to-vigorous physical activity (MVPA) and health-related physical fitness in 12–16-year-old adolescents from south Punjab, Pakistan. Physical activity, according to our findings, is positively correlated with physical fitness indicators, including muscular strength (hand grip, modified pull-ups), cardiovascular endurance (20-m shuttle run test), and aerobic fitness (plank). The results revealed that MVPA is an effective, practical, and accurate predictor of health-related physical fitness in school-aged adolescents. Accordingly, researchers believe moderate-to-vigorous activity plays a critical and necessary role in the physical growth, mental development, and maintenance of health-related physical fitness in adolescents, particularly in the areas of body composition, muscular strength, cardiovascular endurance (CRF), and aerobic fitness. Furthermore, boys were observed to be more dominant in strength, cardiovascular fitness, and endurance overall, but girls were found to have better core muscle strength than boys; results suggested further if both genders turn their lifestyle towards more physically active or more engagement of MVPA in daily life can raise their health-related fitness. As long as gender differences exist, great emphasis should be placed on them, and various interventions may be implemented. Gender-specific MVPA recommendations, guidelines, and health promotion activities targeting adolescents should consider these findings. The findings of this study will help researchers, scientists, physical education instructors, educational officials, and the government design health promotion initiatives that target the risk of physical inactivity among adolescents and holistically promote their physical fitness and well-being.

## Figures and Tables

**Table 1:** Age- and gender-specific demographic analysis.

Age (years)	Boys	Girls	Total
12 N (%)	291 (19.7%)	299 (20.24%)	590 (19.9%)
13 N (%)	295 (19.97%)	298 (20.17%)	593 (20.0%)
14 N (%)	298 (20.17%)	296 (20.04%)	594 (20.0%)
15 N (%)	298 (20.17%)	300 (20.31%)	598 (20.1%)
16 N (%)	295 (19.7%)	300 (20.31%)	595 (20.0%)
Total	1477 (100%)	1493 (100%)	2970 (100%)
Multan N (%)	495 (33.51%)	498 (33.71%)	993 (33.4%)
D G khan N (%)	491 (33.24%)	498 (33.71%)	989 (33.3%)
Bahawalpur N (%)	491 (33.24%)	497 (33.64%)	988 (33.3%)
Total	1477 (100%)	1493 (100%)	2970 (100%)

**Table 2:** Gender-specific descriptive analysis of anthropometric parameters, PF, and MVPA.

Component	Total *x* MVPA (*n* = 2970)	Boys *x* MVPA (*n* = 1477)	Girls *x* MVPA (*n* = 1493)	*p* value
Age (years)	14.01 ± 1.41	14.01 ± 1.41	14.00 ± 1.41	0.927
Height (cm)	159.53 ± 10.51	160.50 ± 11.50	158.57 ± 9.34	<0.001
Weight (kg)	43.00 ± 9.10	45.02 ± 9.78	41.00 ± 7.89	<0.001
BMI (kg/m^2^)	16.80 ± 2.67	17.30 ± 2.41	16.29 ± 2.82	<0.001
HG (kg)	24.39 ± 13.36	31.81 ± 14.85	17.06 ± 5.39	<0.001
PE (sec)	74.87 ± 66.46	80.72 ± 73.48	69.07 ± 58.16	<0.05
MPU (n)	8.78 ± 7.40	12.53 ± 7.74	5.06 ± 4.69	<0.001
SRT (l)	27.15 ± 15.97	31.00 ± 17.00	23.00 ± 14.00	<0.001
MVPA (min)	58.52 ± 36.98	58.05 ± 37.02	58.98 ± 36.95	0.404

Note: the data were presented as mean ± standard deviation. BMI : body mass index; HG (kg): hand grip (kilograms); PE (sec): plank exercise (seconds); MPU (n): modified pull-up (completed number); SRT (l): shuttle run test (completed laps); MVPA : moderate-to-vigorous physical activity (minutes per day).

**Table 3:** Linear regression model relationship between physical fitness and MVPA (*n* = 2970).

Predictors	HG			MPU			*P*E			SRT		
	Β	*R* ^2^	*p*	Β	*R* ^2^	*p*	Β	*R* ^2^	*P*	*β*	*R* ^2^	*p*
MVPA	0.297	0.547	<0.001	0.106	0.411	<0.001	0.894	0.382	<0.001	0.332	0.531	<0.001

Note: dependent variable: HG = hand grip; MPU = modified pull-up; PE = plank exercise; SRT = 20-m shuttle run test.

**Table 4:** Linear regression model relationship between physical fitness and MVPA (boys *n* = 1493).

Predictors	HG			MPU			PE			SRT		
	*β*	*R* ^2^	*p*	Β	*R * ^2^	*p*	Β	*R* ^2^	*p*	*β*	*R* ^2^	*p*
MVPA	0.387	0.577	<0.001	0.151	0.499	<0.001	0.956	0.364	<0.001	0.386	0.556	<0.001

Note: dependent variable: HG = hand grip; MPU = modified pull-up; PE = plank exercise; SRT = 20-m shuttle run test.

**Table 5:** Linear regression model relationship between physical fitness and MVPA (girls, *n* = 1477).

Predictors	HG			MPU			PE			SRT		
	*β*	*R* ^2^	*p*	Β	*R * ^2^	*p*	*Β*	*R* ^2^	*p*	*β*	*R* ^2^	*p*
MVPA	0.21	0.665	<0.001	0.063	0.403	<0.001	0.833	0.413	<0.001	0.279	0.526	<0.001

Note: dependent variable: HG = hand grip; MPU = modified pull-up; PE = plank exercise; SRT = 20-m shuttle run test.

## Data Availability

The authors can also make data available on demand via a data access committee or institutional review board. The corresponding author should be contacted to initiate a request for data access, which should be granted after approval by the Shanghai University of Sport's institution review board in Shanghai, China.
